# Data-Gathering, Belief Flexibility, and Reasoning Across the Psychosis Continuum

**DOI:** 10.1093/schbul/sbx029

**Published:** 2017-03-08

**Authors:** Thomas Ward, Emmanuelle Peters, Mike Jackson, Fern Day, Philippa A Garety

**Affiliations:** 1 Department of Psychology, Institute of Psychiatry, Psychology & Neuroscience, King’s College London, London, UK; 2 NIHR Biomedical Research Centre for Mental Health, South London and Maudsley NHS Foundation Trust, London, UK; 3 School of Psychology, Bangor University, North Wales, UK; 4 Betsi Cadwaladr University Health Board, North Wales, UK

**Keywords:** jumping-to-conclusions, psychotic experiences, need-for-care, experiential and rational reasoning, cognitive models of psychosis

## Abstract

**Background:**

There is evidence for a group of nonclinical individuals with full-blown, persistent psychotic experiences (PEs) but no need-for-care: they are of particular importance in identifying risk and protective factors for clinical psychosis. The aim of this study was to investigate whether reasoning biases are related to PEs or need-for-care.

**Method:**

Two groups with persistent PEs (clinical; *n* = 74; nonclinical; *n* = 92) and a control group without PEs (*n* = 83) were compared on jumping-to-conclusions (JTC) and belief flexibility. A randomly selected subset of interviews (*n* = 104) was analyzed to examine differences in experiential and rational reasoning.

**Results:**

As predicted JTC was more common in the clinical than the other 2 groups. Unexpectedly no group differences were observed between clinical and nonclinical groups on measures of belief flexibility. However, the clinical group was less likely to employ rational reasoning, while the nonclinical group was more likely to use experiential reasoning plus a combination of both types of reasoning processes, compared to the other 2 groups.

**Conclusions:**

Reasoning biases differ in groups with PEs with and without need-for-care. JTC is associated with need-for-care rather than with PEs. The ability to invoke rational reasoning processes, together with an absence of JTC, may protect against pathological outcomes of persistent PEs. However, marked use of experiential reasoning is associated with the occurrence of PEs in both clinical and nonclinical groups. Implications for theory development, intervention and further research are discussed.

## Introduction

There is growing evidence for the existence of a group of nonclinical individuals with full-blown, persistent psychotic experiences (PEs) but no need-for-care.^1^ These are individuals with persistent, nondistressing PEs, and they show few signs of distressing delusions or paranoia.^1^ They also show a flexible range of benign (typically normalizing, often spiritual) appraisals of their anomalous experiences^2,3^; they do not show the threat-related, maladaptive appraisals commonly seen in clinical groups.^4,5^ As such this group is of particular importance in identifying risk and protective factors for clinical psychosis.

One protective factor that may distinguish this unique group is an absence of the reasoning biases associated with maladaptive appraisals of anomalous experience. Individuals with distressing delusions, specifically, make decisions on the basis of limited evidence in probabilistic reasoning tasks: the so-called “jumping-to-conclusions” (JTC) data-gathering bias.^6^ JTC is exacerbated in acute states, and may represent a trait vulnerability^7^ and a prognostic marker.^8^ Work on JTC has led to investigations into belief flexibility, defined as the metacognitive ability to reflect on one’s own beliefs,^6^ and the related bias against disconfirmatory evidence (BADE),^9,10^ defined as the neglect of counterarguments for decision-making. Lack of belief flexibility is commonly reported in people with clinical delusions, with typical rates ranging between 50% and 75%,^6^ and is a related but distinct process to JTC and belief conviction.^11^ To date, belief flexibility has been studied almost exclusively in the context of clinical delusions. The flexible range of benign *explanations of experiences* observed in individuals with persistent nonclinical PEs, make JTC and belief flexibility constructs of interest for understanding the absence of threat appraisals in this group.^4,5^

The definition of belief flexibility (ie, an ability to step back, consider the possibility of being mistaken and reflect on alternative explanations) overlaps with the analytic, controlled “type 2” reasoning identified in dual-process models of cognition.^12^ Recent investigations of reasoning and psychosis have drawn on Epstein’s cognitive-experiential self-theory (CEST) and associated nomenclature of “experiential/ intuitive” (emotion based) and “rational” systems.^13^ Modest positive correlations have been found between experiential reasoning and paranormal and superstitious beliefs and schizotypy, with rational reasoning showing the converse relationship.^14–16^ Freeman et al^16^ also found that a perceived reliance on experiential reasoning is associated with paranoid thinking in the general population, while reliance on deliberation (rational reasoning) is associated with fewer paranoid thoughts. These studies point to the relevance of dual-process models to the psychosis continuum; in particular suggesting that rational reasoning may be associated with reduced paranoia while over-reliance on experiential (emotion-based) reasoning may be associated with unusual beliefs and paranoia across the psychosis continuum.

In summary, studying individuals with persistent nondistressing PEs with no need-for-care can illuminate protective factors mitigating the risk of transition to clinical psychosis. It is important to distinguish this group from individuals with an “at risk mental state” (ARMS) and those in the general population scoring highly on measures of delusion-proneness, on the lower end of the continuum of risk of psychosis, where elevated paranoia would be expected and evidence of reasoning biases would be predicted.^7,17,18^ We hypothesize that these groups will differ with respect to reasoning biases; and more specifically that the absence of the clinically established biases in JTC and belief flexibility, together with differences in dual-process reasoning may help to explain the adaptive meaning-making in this unique group.

## Aims

The primary aim was to investigate whether JTC and belief flexibility are associated with need-for-care rather than PEs alone: individuals with persistent PEs, with and without need-for-care, and a control group without PEs, were compared on responses to standardised assessments of JTC and belief flexibility. A secondary aim was to compare how the three groups reason about their PEs/a personally meaningful belief, applying a dual-process framework (detailed in the “Methods” section).

## Hypotheses

1. The clinical group will be more likely to show JTC than nonclinical and control groups, who will not differ from each other.
2. The nonclinical and the control groups will be more likely to demonstrate belief flexibility about their own beliefs and a control belief, than the clinical group.
3. JTC will be associated with belief inflexibility (possibility of being mistaken) across the sample (replicating^6^).
4. The nonclinical group will score more highly than the clinical group on rational reasoning. Group differences in experiential reasoning will be explored.

## Methods

This was a planned study within the UNIQUE study.^1^

### Participants

Three groups were recruited across 2 sites (Urban—South London; Rural—North Wales): (1) patients with PEs (diagnoses F20–F39) (clinical group; *n* = 74); (2) individuals with PEs without need-for-care (nonclinical group; *n* = 92); (3) controls with no PEs (*n* = 83), matched to nonclinical group in age, gender, ethnicity, and education.

Exclusion (all groups): (1) age <18; (2) insufficient English; (3) neurological history, head injury, epilepsy; (4) primary substance dependence; (5) estimated IQ <70 (10 individuals otherwise meeting criteria for the clinical group had estimated IQ below 70 and were excluded on the basis that reasoning task performances may not be valid.)

Main inclusion criterion for clinical/nonclinical groups was evidence of current PEs (past month/ in clear consciousness), scoring ≥2 (“occasional”) on at least one item of the Scale for the Assessment of Positive Symptoms (SAPS^19^). Specific inclusion criteria for nonclinical group: (1) experiences started ≥5 years previously (avoiding possibly prodromal individuals); (2) did not score 2 (“unmet need”) on basic self-care and “psychological distress” items (relating to PEs) of Camberwell Assessment of Need Short Appraisal Schedule (CANSAS^20^); (3) no previous contact with mental health services/general practitioner (GP) regarding PEs (nor anyone on their behalf); (4) no previous contact with specialist mental health provision not available through general/family practitioner; (5) judged by research worker to not be in need-of-care for PEs. A specific exclusion criterion for controls was endorsement of any unusual experience item at screening; see^1^ and online supplement.

### Measures

#### Appraisals of Anomalous Experiences Interview (AANEX^2^).

A semi-structured interview, eliciting current PEs, and associated emotional/cognitive correlates. Part one (AANEX-Inventory, short form^21^) comprises 17 anomalous experiences (including hearing voices, somatic experiences, experiences of reference, thought/mind permeability), rated on a 3-point scale (1 = not present; 2 = unclear; 3 = present; range 17–51) over lifetime, and currently (past month). Inter-rater reliabilities (IRR; *N* = 35; 16 clinical, 19 nonclinical) indicated almost perfect agreement (intra-class correlation coefficient (ICC) > .8): total (ICC = .995); current (ICC = .997); lifetime (ICC = .998). Experiences elicited by AANEX-Inventory anchored Part two (AANEX-CAR; context, appraisals and response), including belief flexibility items.

#### JTC Bias—The Beads Task.

JTC was assessed using a computerised (difficult; 60:40) version of the data-gathering beads task.^6^ Number of beads drawn prior to decision (maximum 20) was recorded. JTC has been operationalised as a decision made after ≤2beads.^6^ Decision conviction and accuracy were recorded.

#### Belief Flexibility.

Assessed using 2 standardized, widely used items^6,11^: (1) possibility of being mistaken^22^ and (2) alternative explanations.^23^ Both are scored yes/no indicating presence/absence of flexibility. For clinical and nonclinical groups the anchor was the main belief about the cause of PEs (ie, flexibility was assessed in the context of explanations of experiences rather than specific delusions, as in earlier studies). Illness/biological attributions were treated in an identical fashion to other explanations, that is, individuals were asked whether they might be mistaken/to generate alternative beliefs. Controls were presented with five beliefs in order to identify a comparable personally significant belief^24^ about a fundamental issue (*God exists; There is an afterlife; The climate is changing due to human factors; Everything happens for a reason; There is a higher force at work in the world*). They were asked to select the item that was “*...most important to the way in which you think about the world- you can either agree or disagree with the statement you choose- the important thing is that the statement is one that matters personally to you.*” If no item reached a 70% personal significance threshold, they were asked to generate their own belief (relating to the physical or metaphysical nature of the world or the functioning of society; purely value-based judgments/opinions were excluded given methodological issues identified in a previous study (Belief flexibility data were only available for 45 of the control group. This comprised 18 males (40.0%) and 27 females; mean age of 47.4 years (range 21–73); 40 (88.9%) self-identified as “white” background; 35 (77.9%) were in employment/education/training. There were no significant differences between those that provided belief flexibility data compared to those that did not on any key demographic or JTC reasoning variable.).^24^

Belief flexibility was also assessed in all groups in relation to a control belief: “the sun will rise tomorrow.”^24,25^ IRR for main belief items (*n* = 45; 16 clinical, 19 nonclinical, 10 control) demonstrated at least substantial agreement (ie, kappas >.6 following Landis and Koch^26^; “Possibility of being mistaken” = 0.77; “Alternative Explanations” = 0.86.)

#### Ratings of Experiential Vs Rational Reasoning Processes.

A novel method of rating dual-process reasoning was developed. Responses to belief flexibility and AANEX-CAR^2^ appraisal (“*what sense do you make of your experiences?*”) items were transcribed for analysis, since they elicited statements reflecting reasoning processes. Definitions of rational and experiential reasoning were developed using Epstein’s CEST framework,^13,27^ following Freeman et al.^16^ Definitions (see Appendix) were closely tied to recent integrative dual-process theories.^28^ Interviews were scored 0–2 (0 = *no evidence of reasoning process*; 1 = *questionable/possible use*; 2 = *clear and spontaneous use*). Ratings (clinical and nonclinical groups) were based on explanations of PEs. In the clinical group these included (in addition to de facto delusions), biological/biomedical explanations and spiritual explanations that showed clear overlap with the nonclinical group. The focus on the ***process*** of reasoning rather than belief content mitigates concerns over tautology in particular given that ratings could not be blind to belief content. IRR was conducted on 21 (20% of analysed sample) transcripts (6 clinical, 7 nonclinical, 8 control) with an independent blinded rater; agreement for rational (kappa = 0.67) and experiential (kappa = 0.71) reasoning fell into the “substantial agreement” (0.6–0.8) range.^26^

#### Wechsler Adult Intelligence Scale 3rd Edition—Short Form (WAIS-III^29^).

One subtest of each index was administered: information (verbal comprehension), block design (perceptual organization), arithmetic (working memory), and digit symbol (processing speed). Scores were prorated to estimate IQ.

### Procedures

#### Ethical Approval.

NRES Committee London–Westminster (Ref: 12/LO/0766), South London & Maudsley/Institute of Psychiatry (SLAM/IoP) R&D Office (Ref: R&D2012/047), and BCUHB R&D Office (Reference: Jackson/LO/0766). Participants were screened over the phone or in person. Eligible participants completed all assessments, plus other measures and experimental tasks (not reported in this article), and given an honorarium.

### Statistical Analysis

Data were analyzed using SPSS version 20. Normally distributed continuous variables were analyzed using *t*-tests and 1-way ANOVAs. Where deviations from a normal distribution could not be corrected by transforming, nonparametric testing was conducted. Group differences in JTC (beads drawn) were analyzed using Mann–Whitney (individual group comparisons) and Kruskall–Wallis (3-group comparisons) tests. χ^2^ tests were conducted on dichotomised variables: JTC extreme responding (decision after ≤2 beads), flexible versus nonflexible responses to belief flexibility items and, for experiential/rational ratings, “*clear and spontaneous use of specific reasoning processes*” (a maximum rating of 2) vs “*no*” or only “*questionable evidence*” (a rating of 0 or 1).

The view that ANCOVA (analysis of covariance, commonly employed in psychological research) or equivalent methods should be used to achieve the goal of “controlling for” real group differences has been condemned.^30^ We therefore report the group differences on our hypothesized variables without including as covariates in the analysis established risk factors for psychosis, on which the groups differ (such as IQ, ethnicity, and gender) or those inherent to need-for-care status (eg, impaired functioning, anxiety, depression, etc).

## Results

### Demographics and Clinical Data

The groups did not differ in age (table 1). In line with previous research, the nonclinical group was more likely to be female, and nonclinical and control groups were less likely to belong to black or minority ethnic groups, had higher IQ, and were more likely to be in education/employment/training. The nonclinical group had a younger age of onset of their PEs than the clinical group, with 77.2% reporting voices during their lifetime. Overall they were less symptomatic than the clinical group on the SAPS^19^ and SANS,^31^ although not significantly different on the AANEX,^2^ a measure designed to assess anomalous experiences across the psychosis continuum. The nonclinical group experienced hallucinations in all modalities as well as first-rank symptoms, but scored lower on global delusions (with minimal endorsement of paranoid or grandiose delusions). Ideas of reference were the most commonly rated delusion in the nonclinical group, but these were still less common than in the clinical group. The nonclinical group also reported fewer cognitive difficulties and negative symptoms (see^1^ for further information).

**Table 1.** Demographic and Clinical Data by Group

	Clinical (*n* = 74)	Nonclinical (*n* = 92)	Controls (*n* = 83)	Significance tests
London:Bangor	35:39	51:41	43:40	
Female (%)	25 (33.8%)	67 (72.8%)	57 (68.7%)	χ^2^(2) = 30.1, *P* < .001
Mean age (range)	43 (20–78)	46 (18–80)	46 (21–76)	F_(2,246)_ = 1.142, *P* = .321
Ethnicity				χ^2^(2) = 11.2, *P* = .004 (white vs BME)
White	71.6%	87.0%	90.4%	
Black	21.6%	6.5%	3.6%	
Dual Heritage	2.7%	3.3%	2.4%	
Asian	2.7%	2.2%	2.4%	
Other	1.4%	1.1%	1.2%	
In education/training/employment	18.9%	69.6%	78.3%	χ^2^(2) = 65.2, *P* < .001
Mean IQ (SD)	89^e^ (11.7)	105^b^ (14.0)	112^a^ (16.5)	*F*_(2,225.5)_ = 54.2, *P* < .001
Median psychiatric admissions (range)	4^d^ (0–20)	N/A	N/A	
On anti-psychotic medication	89%	N/A	N/A	
Typical	10%			
Atypical	55%			
Clozapine	24%			
>1 antipsychotic	16%			
% maximum daily dose; median (range)	50 (12–100)^g^			
Mean age at start of psychotic experiences (SD)	22 (10.8)	15 (12.3)	N/A	*t*_(164)_ = 3.8, *P* < .001 (*C>NC*)
Lifetime voices	87.8%	77.2%	N/A	χ^2^(1) = 3.150, *P* = .08
SAPS total	26.4 (15.4)^a^	12.2 (7.2)^a^	N/A	*P < .001
SAPS hallucinations global rating	3.1 (1.9)	2.4 (1.3)	N/A	*P = .001
SAPS delusions global rating	3.6 (1.2)	2.3 (1.4)	N/A	*P < .001
Frequency scoring ≥3 (global delusions)	59/73	37/92	N/A	
Frequency scoring ≥3 (persecutory delusion)	29/73	1/92		
SAPS bizarre behaviour global	0.8 (1.2)	0.1 (0.4)	N/A	*P < .001
SAPS thought disorder global rating	0.9 (1.2)	0.1 (0.3)	N/A	*P < .001
SANS total	20.2 (11.8)^f^	3.0 (3.3)^c^	N/A	*P < .001
SANS global ratings total (sum of 5 global ratings)	8.5 (3.7)	1.5 (1.7)^a^	N/A	*P < .001
SAPS somatic/tactile hallucinations	1.4 (1.8)	2.1 (1.7)	N/A	*P* = .005
SAPS delusions of reference	2.7 (1.7)	1.7 (1.7)	N/A	*P* < .001
SAPS visual hallucinations	1.2 (1.7)	1.6 (1.7)	N/A	*P* = .094
SAPS thought insertion	1.8 (1.9)	1.6 (1.7)	N/A	*P* = .551
SAPS auditory hallucinations	2.7 (2.2)	1.4 (1.4)	N/A	*P* < .001
SAPS mind reading	1.7 (1.9)	1.1 (1.4)	N/A	*P* = .047
SAPS olfactory hallucinations	0.5 (1.1)	0.7 (1.2)	N/A	*P* = .096
SAPS feelings of being controlled	0.9 (1.7)	0.5 (1.1)	N/A	*P* = .093
SAPS voices commenting	1.6 (2.1)	0.3 (1.0)	N/A	*P* < .001
SAPS thought broadcast	1.5 (2.0)	0.2 (0.6)	N/A	*P* < .001
SAPS voices conversing	1.1 (1.8)	0.2 (0.6)	N/A	*P* < .001
SAPS grandiose delusions	0.7 (1.4)	0.2 (0.7)	N/A	*P* = .007
SAPS thought withdrawal	0.7 (1.4)	0.1 (0.5)	N/A	*P* < .001
SAPS religious delusions	0.7 (1.4)	0.1 (0.4)	N/A	*P* < .001
SAPS persecutory delusions	1.9 (1.6)	0.1 (0.4)	N/A	*P* < .001
SAPS inappropriate affect	0.3 (0.9)	0.03 (0.3)	N/A	*P* = .006
SAPS delusions of jealousy	0.3 (0.7)	0.01 (0.1)	N/A	*P* < .001
SAPS delusions of sin/ guilt	0.7 (1.3)	0.01 (0.1)	N/A	*P* < .001
SAPS somatic delusions	0.3 (0.9)	0.01 (0.1)	N/A	*P* = .001
AANEX total current	30.1 (6.2)^b^	28.6 (5.1)	N/A	*t*_(135.0)_ = 1.327, *P* = .19
AANEX total lifetime	36.6 (6.6)^a^	34.8 (4.9)	N/A	*t*_(130.3)_ = 1.935, *P* = .06

Superscript letters represent the number of missing participants as follows: a = 1, b =2, c=3, d = 5, e = 6, f = 7, and g = 10.

*Mann–Whitney tests (all SAPS and SANS scores).

Hypothesis 1: The clinical group will be more likely to show the JTC bias than nonclinical and control groups, who will not differ from each other.

As predicted the clinical group was significantly more likely to demonstrate JTC, both in terms of number of beads drawn and percentage showing extreme responding, compared to the other 2 groups, with small to medium effect sizes (table 2).

**Table 2.** Number of Beads Drawn, Extreme JTC Responding, Conviction and Accuracy on the Beads Task in the 3 Groups

	Clinical (*n* = 74)	Nonclinical (*n* = 92)	Controls (*n* = 83)	Significance Test	Effect sizes/odds ratios (95% CIs)
Mean number of beads drawn (SD); median (IQR)	7.01 (6.06); 5 (1–11)^a^	10.36 (6.60); 10 (5–15)^b^	11.19 (6.09); 11 (8–16)	H(2) = 17.41, ***P* < .001**	
				C < NC; U = 2312.5, ***P* = .002**	***r* = −.246**
				NC = CON; U = 3467, *P* = .345	*r* = −.072
				C< CON; U = 1844.0, ***P* < .001**	***r* =−.325**
Mean of conviction in final choice (SD); median (IQR)	63.45 (27.21); 60 (50–80)^a^	61.02 (25.67); 60 (50–80)^a^	51.83 (27.27); 60 (40–70)^b^	H(2) = 6.175, ***P* = .046**	
				C = NC; U = 3003.50, *P* = .588	*r* = .043
				NC > CON; U = 3019.00, ***P* = .049**	***r* = −.150**
				C > CON; U = 2297.50, ***P* = .024**	***r* = −.183**
*N* (%) extreme responders (≤2)	26 (35.1%)	17 (18.5%)	9 (10.8%)	χ^2^(2) = 14.48, ***P* = .001**	
				C > NC; χ^2^(1) =5.928, ***P* = .015**	**OR = 2.390 (1.174, 4.864**)
				NC = CON; χ^2^(1) = 2.011, *P* = .156	OR = 1.864 (0.781, 4.446)
				C > CON; χ^2^(1) = 13.326, ***P* < .001**	**OR = 4.454 (1.922, 10.322**)
*N* (%) incorrect	16^a^ (22.5%)	12^a^ (13.5%)	9^b^ (10.8%)	χ^2^(2) = 4.38, *P* = .112	

The effect size, *r*, is calculated following (Rosenthal, 1991) by the equation $r=Z/\sqrt{N}$, where *Z* is the *z*-score and *N* is number of observations on which *z* is based. For *r* as an effect size .1 = small, .3 = medium, .5 = large.

^a^Three missing data points.

^b^One missing data point.

Hypothesis 2: The nonclinical and the control groups will be more likely to demonstrate belief flexibility about their own beliefs and a control belief, than the clinical group.

The hypothesis was not supported, with no differences between the clinical and nonclinical groups on belief flexibility items on either the main or control belief (table 3). For the main belief, there was a trend (*P* = .057) toward the nonclinical group showing less flexibility on the “possibility of being mistaken” item compared to the control group.

**Table 3.** Belief Flexibility Items for Main Belief and Control Belief

Main belief	Clinical (*n* = 71)	Nonclinical (*n* = 92)	Controls (*n* = 40^1^)	Significance Tests	Odds Ratio (95% CIs)
Personal significance (SD)	N/A	N/A	85.63 (9.62)	H(2) = 4.015, *P* = .134	
Mean of conviction (SD); Median (IQR)	75.65^a^ (25.14); 80 (60–99)	82.76 (21.24); 90 (76–100)	80.52 (19.55); 85 (70–99)		
PM?				Overall: χ^2^(2) = 3.689, *P* = .158	
No	32	44	12	C vs NC; χ^2^(1) = .186, *P* = .666	1.145 (0.617, 2.128)
Yes	40	48	28	CON vs NC; χ^2^(1) = 3.627, *P* = .057	2.139 (0.970, 4.714)
% Yes	55.6	52.2	70.0	CON vs C; χ^2^(1) = 2.249, *P* = .139	1.867 (0.822, 4.241)
AE?				Overall: χ^2^(2) = 3.033, *P* = .219	
No	35	38	13	NC vs C; χ^2^(1) = 1.035, *P* = .309	1.382 (0.741, 2.577)
Yes	36	54	27	CON vs NC; χ^2^(1)=.912, *P* = .340	1.462 (0.669, 3.192)
% Yes	50.7^b^	58.7	67.5	CON vs C; χ^2^(1) = 2.941, *P* = .086	2.019 (0.899, 4.534)
**Control belief**	**Clinical (*n* = 73**)	**Nonclinical (*n* = 92**)	**Controls (*n* = 45**)		
Mean of conviction (SD); Median (IQR)	89.04 (23.14); 100 (98.5–100)	93.92 (16.16); 100 (99–100)	97.15 (9.50); 100 (99–100)	H(2) = 0.268, *P* = .874	
PM?				Overall: χ^2^(2) = 0.461, *P* = .794	
No	37	45	20	C vs NC; χ^2^(1) = 0.025, *P* = .875	1.051 (0.567, 1.945)
Yes	36	46	25	CON vs NC; χ^2^(1) = 0.302, *P* = .582	1.223 (0.597, 2.506)
% Yes	49.3	50.5^b^	55.6	CON vs C; χ^2^(1) = 0.434, *P* = .510	1.285 (0.609, 2.708)

PM, “possibility of being mistaken”; AE, “alternative explanations.”

^1^5/45 were excluded because no belief was rated above the 70% threshold.

^a^Two missing data points.

^b^One missing data point.

^c^Three missing data points.

Hypothesis 3: JTC will be associated with belief inflexibility (possibility of being mistaken) across the sample.

The hypothesis was not supported: people with JTC (extreme responding) were not more likely to show belief inflexibility on either main (χ^2^(1) = 0.585, *P* = .445; OR [95% CI] = 1.288 [0.673, 2.466]) or control (χ^2^(1) = 0.464, *P* = .496; OR [95% CI] = 1.249 [0.658, 2.372]) beliefs.

Hypothesis 4: The nonclinical group will score more highly than clinical group on rational reasoning.

**Fig. 1.**
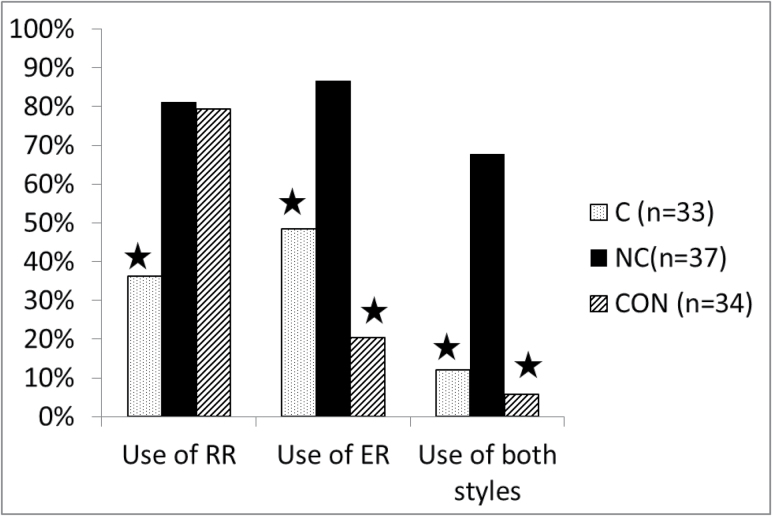
Percentage in each group showing clear evidence of rational reasoning (RR), experiential reasoning (ER) and both ER and RR (stars indicate differences at *P* ≤ .001 between adjacent groups). C, clinical; NC, nonclinical; CON, controls.

The nonclinical group was more likely than the clinical group to receive a maximum rating (“clear consistent evidence”) for rational reasoning, while the nonclinical and control group did not differ (table 4 and figure 1). The nonclinical group was more likely to show experiential reasoning than the clinical group, who in turn was more likely to employ it than the control group. Finally in terms of use of a combination of both styles of reasoning, the nonclinical group was more likely to show “clear consistent evidence” of a combination of both styles of reasoning than the other 2 groups.

**Table 4.** Group Differences in Use of Rational Reasoning (RR), Experiential Reasoning (ER), and Both Styles of Reasoning

	C (*n* = 33)	NC (*n* = 37)	CON (*n* = 34)	Significance Tests	Effect Sizes/Odds Ratios (95% CIs)
Use of RR? *n* (%)	12/33 = 36.4%	30/37 = 81.1%	27/34 = 79.4%	χ^2^(2) = 19.484, ***P* < .001**	
				C vs NC; χ^2^(1) = 14.533, ***P* < .001**	**OR = 7.500 (2.531, 22.223**)
				NC vs. CON; χ^2^(1) =.031, *P* = .860	OR = 0.900 (0.279, 2.899)
				CON vs C; χ^2^(1) = 12.757, ***P* < .001**	**OR = 6.750 (2.263, 20.132**)
Use of ER? *n* (%)	16/33 = 48.5%	32/37 = 86.5%	7/34 = 20.6%	χ^2^(2) = 31.256, ***P* < .001**	
				C vs NC; χ^2^(1) = 11.688, ***P* = .001**	**OR = 6.800 (2.124, 21.774**)
				NC vs CON; χ^2^(1) =31.079, ***P* < .001**	**OR = 24.390 (7.042, 83.333**)
				CON vs C; χ^2^(1) =.781, ***P* = .016**	**OR = 3.636 (1.238, 10.638**)
Use of ER and RR? *n* (%)	4/33 = 12.1%	25/37 = 67.6%	2/34 = 5.9%	χ^2^(2) = 39.450, ***P* < .001**	
				C vs NC; χ^2^(1) =22.099, ***P* < .001**	**OR = 15.104 (4.320, 52.805**)
				NC vs CON; χ^2^(1) =28.608, ***P* < .001**	**OR = 33.333 (6.849, 166.666**)
				CON vs C; χ^2^(1) = 0.799, *P* = .371	OR = 2.208 (0.376, 12.987)

### Additional Exploratory Analysis

Individuals showing clear evidence of rational reasoning were more likely to show higher belief flexibility on both the “possibility of being mistaken” (χ^2^(1) = 4.234, *P* = .040; OR [95% CI] = 2.375 [1.033, 5.459]) and “alternative explanations” items (χ^2^(1) = 6.936, *P* = .008; OR [95% CI] = 3.048 [1.311, 7.083]). This was expected given definitional overlap and use of belief flexibility items for ratings of rational reasoning. Individuals showing extreme JTC responding were less likely, at trend level, to use rational reasoning (χ^2^(1) = 3.339, *P* = .068; OR [95% CI] = 2.417 [0.924, 6.322]). Individuals showing clear evidence of experiential or “both rational and experiential” reasoning did not show significant differences on any of the other reasoning variables (see supplementary material).

For reference, additional analysis involving IQ and the key reasoning variables are presented in supplementary materials. Overall, IQ (when entered alongside group) significantly predicted JTC and some (but not all) of the other reasoning variables; correlation with beads drawn was low (*r* = .260, *P* < .01) indicating a small effect. Gender was not a significant predictor of any dependent variable and the addition of gender to regression models had no effect on the results.

## Discussion

This study investigated whether the established reasoning biases of JTC and belief flexibility are associated with presence of PEs or with need-for-care status, by comparing individuals with persistent PEs, with and without need-for-care. It also applied, for the first time, a dual-process framework to investigate the process of explaining (reasoning about) PEs. Reasoning biases were found to differ in people with persistent PEs with and without need-for-care. The results suggest that an ability to invoke rational reasoning processes, together with an absence of limited data gathering (JTC), may protect against the development of need-for-care when persistent PEs are present, while marked use of experiential reasoning is associated with the presence of PEs.

The novel finding that JTC is not associated with the presence of full-blown PEs in the absence of a need-for-care, offers a new perspective on the causal role of this reasoning bias in the context of PEs. Consistent with the hypothesized role of reasoning biases in cognitive models of psychosis,^32,33^ we suggest that absence of JTC reduces the likelihood of developing maladaptive threat appraisals and the resultant characteristic paranoia observed in clinical populations.^4,5^ While the specificity of JTC to delusions remains subject to debate, a recent well-conducted meta-analysis has concluded that there is evidence for specificity of JTC to delusions.^34^ Since the unique nonclinical group in this study is characterized by persistent nondistressing PES in the absence of paranoia/distressing delusions, the absence of JTC is consistent with specificity to distressing delusions and not hallucinations.^35,36^

As also hypothesized, the clinical group was less likely than the other 2 groups to employ rational reasoning when explaining their PEs. Thus, those whose PEs were associated with a need-for-care, employed less “type 2,” reflective reasoning; this type of reasoning assists with generating alternative, less distressing, appraisals of these experiences. In contrast, the nonclinical group (with PEs but without need-for-care) did not differ from controls in rational reasoning, and was equally likely to be reflective; consistent with the association between rational reasoning and reduced paranoia in the general population, suggesting an adaptive role for such reasoning.^16^ However, the nonclinical group did also show higher levels of experiential, “gut” reasoning: over 60% showed clear evidence of both reasoning processes. Consistent with these findings, Daalman and colleagues^37^ found that healthy voice-hearers showed high levels of “emotional reasoning” (a bias showing clear overlap with experiential reasoning) and Wolfradt and colleagues^14^ reported that a preference for ***both*** intuitive (“type 1”) and rational (“type 2”) thinking styles leads to stronger paranormal beliefs.

Belief flexibility findings were less clear-cut. While there was some evidence of the nonclinical group being less likely to accept the possibility of being mistaken than controls, there were no differences between the clinical and nonclinical group on any item. The rates of flexibility reported on the “possibility of being mistaken” item are comparable with previous studies^6,11^; however the focus on explanations of experiences rather than delusional beliefs per se, may account for the higher than expected flexibility in the clinical group on the “alternative explanation” item compared to earlier studies.^11,23^ The only previous study comparing delusions with personally significant beliefs^24^ reported no differences in flexibility on the main belief between deluded, remitted, and control groups (consistent with current findings), but found evidence of greater flexibility in controls on the control/standard belief. The current study may be considered an improvement in terms of larger sample size and more stringent criteria for selection of comparison belief for controls.

The dual-process ratings, developed for this study, offer a novel way of assessing “online” reasoning in an ecologically valid manner. Rational reasoning was associated with belief flexibility and, at trend level, with absence of JTC, providing some evidence of convergent validity. However, the apparent disparity between explicit belief flexibility responses and the use of rational reasoning in the nonclinical group (evidenced by the dual-process ratings) suggests that while responses to standard belief flexibility questions can predict positive engagement with psychological therapy,^11,38,39^ these items may be less successful in elucidating subtle real-world reasoning processes.

The study findings need to be interpreted in the context of certain methodological limitations. The cross-sectional nature of the study means the possibility cannot be excluded that associations, for example, between JTC and need-for-care, may be epiphenomenal to, or a consequence of, need-for-care, or relate only to specific aspects (eg, presence of delusions or other variables, such as IQ, on which the groups differ). The current study did not set out to examine the role of neurocognition in need-for-care status and its contribution to these reasoning biases. In future, using a comprehensive battery, we would recommend further examination of the relationship between IQ, neurocognitive deficits, and belief flexibility (and dual-process reasoning), as has been done previously with the JTC bias.^40^ The novel dual-process findings should also be considered preliminary since ratings were conducted on only a subset of the overall sample and they require replication in a larger, fully-blinded sample. Future work should take into account the possibility that clinical groups may engage in less reasoning overall (regardless of type) when compared to other groups^41^ although this issue was mitigated in the current study by ratings being conducted in terms of presence of one clear example of experiential or rational reasoning rather than frequency of use. The fact that some differences on belief flexibility did not reach statistical significance may be attributable to reduced power given the relatively small sample size for these analyses specifically.

In summary, this study has found that reasoning biases differ in groups with PEs with and without need-for-care. People with persistent PEs but without need-for-care did not JTC and made more use of rational reasoning than those with need-for-care. Therefore, absence of JTC and an ability to invoke rational reasoning represent plausible protective factors within this unique group. In contrast, a high use of experiential reasoning, common to both PE groups, may play a role in the occurrence of both benign and clinically distressing PEs. It is notable that dual-process models of reasoning are starting to inform a new generation of targeted reasoning interventions, focused on constructs such as “fast” and “slow” thinking, with promising results.^39,42–45^ The dynamic nature of real-world reasoning is likely to involve a complex interplay of processes competing and combining to produce observed behavior.^28^ Gaining a more nuanced understanding of how individuals with PEs, with and without a need-for-care, reason about their experiences will assist in refining future developments in clinical interventions for distressing psychosis.

## Supplementary Material

Supplementary data are available at *Schizophrenia Bulletin* online.

## Funding

This study was supported by Medical Research Council (Reference: G1100568).

## Supplementary Material

Supplementary Materials

## Appendix 1: Definitions of Reasoning Processes

### Experiential reasoning scoring

#### Type 1 process: fast, high capacity, independent of working memory and cognitive ability

Reasoning processes to be rated as experiential in cases of:

1. (Main criteria)—Reasoning clearly tied to affect (feeling, hunches, gut-feelings, vibes, etc) that is, reasoning on basis of internal subjective states.
2. Where there is evidence of judgements being made in a rapid, nondeliberative manner
  - For example, “flashes of understanding/ insight,” “sudden revelations,” “sudden shifts in understanding.”
3. Where the person expresses the view that their way of understanding cannot easily be reduced into words/logic.
  - Where the individual states something akin to “I just know”
  - Where the individual states something akin to “I don’t know if I can describe what I mean”
  - Where an individual states limitations to rationality, logic (“some things science can’t understand,” etc).

### Rational reasoning processes

#### Type 2 process: slow, low capacity, heavily dependent on working memory and related to individual differences in cognitive ability].

Reasoning processes to be rated as rational reasoning on the basis of:

1. Evidence of slower, deliberative processing
  - describing a process of reflection/analyzing over a period of time
  - For example, “I have thought long and hard about this”
2. Where the person acknowledges applying a logical, rational approach to developing their understanding.
  - Examples such as “I thought about this, considered other possibilities, checked “x” out, asked others,” etc.
3. Where the person draws on evidence collected from or (or at least available to) other people, that is, third-person proof/evidence.
4. Where the person acknowledges how they were (at any stage) “not certain/ in doubt” about belief.
  - For example, “I wasn’t sure, it was hard to work out what was going on at the start,” “I’m still not sure.”
5. Specific use of any of the following
  - Hypothetical reasoning (“if . . . then”)—consistent with a logical reasoning process.
  - Spontaneous use of “cognitive simulation,” that is, the person showing an ability to “decouple” from their own understanding to acknowledge other ways of understanding the same experiences.

Explicit use of probability estimates on the basis that this stands opposed to the idea of “just knowing.”
