# Supplementary material for: Data-Gathering, Belief Flexibility, and Reasoning Across the Psychosis Continuum
Source: Schizophr Bull. 2017 Mar 8;44(1):126–36. doi: 10.1093/schbul/sbx029 (PMC5768047; doi:10.1093/schbul/sbx029)
Supplement: Supplementary Materials [file sbx029_suppl_supplementary_materials.doc]

**Supplementary information**

***Recruitment:***

Recruitment of the clinical group took place from both inpatient (N=22) and community (N=52) services of the South London and Maudsley NHS Foundation Trust (SLaM) and Betsi Cadwaladr University Health Board (BCUHB). The non-clinical group were predominantly recruited using our previous sampling strategy (Heriot-Maitland, Knight, & Peters, 2012; Ward, et al., 2014) targeting specialist sources such as psychic and spiritualist fora, mediums, and other special interest websites. Additional recruitment was conducted via an epidemiologically representative community sample (South East London Community Health Study: SELCoH; Hatch et al., 2011) and GP registers selected from the same geographical area as our South London clinical sample. The control group were matched to the non-clinical group in age, gender, ethnicity, and education level and recruited from advertisements in various community settings such as GP registers and university research registers and circulars, or were volunteered by non-clinical participants (see Peters et al., 2016, for further details).

Individuals in the non-clinical and control group who had received diagnoses of, and/or treatment for, common mental health problems (such as anxiety and depression), or had been in contact with primary care services for issues unrelated to their psychotic experiences, were included in the study, meaning that the definition of ‘need-for care’ in the current study is more specifically ‘need-for-care in the context of psychotic experiences’. Specific exclusion criterion for the control group was endorsement of any unusual experience item at screening (see below).

***Screening:***

The clinical group were screened through clinicians and/or case-note review. The non-clinical and control group were screened with the Unusual Experiences Screening Questionnaire (UESQ; derived from the AANEX-Inventory (Brett, et al., 2007) and the Psychosis Screening Questionnaire (PSQ; Bebbington & Nayani, 1995)*.* As well as no endorsement of any item on these measures, controls scored below one standard deviation of the mean on the Unusual Experiences (UnEx) subscale (i.e. a cut-off score of 15) of the Oxford-Liverpool Inventory of Feelings and Experiences (O-LIFE; Mason & Claridge, 2006). Further information on the screening of participants is provided in Peters et al. (2016).

***Detailed information on available data for controls for Belief Flexibility analysis:***

As noted above the current study was conducted as part of the larger UNIQUE study (Peters et al, 2016). In the original UNIQUE study design belief flexibility items were only administered to the clinical and non-clinical groups, on the basis that items pertained to ‘explanations of unusual experiences’. However, in finalising the design of this subsidiary study it became clear that presenting data on belief flexibility in the control group would strengthen the study. Therefore all controls who had participated in the original study (n=83) were re-contacted with 45 out of the 83 people agreeing to return to complete these additional belief flexibility items. Belief flexibility data were therefore only available for 45 of the Control group. This subset comprised 18 males (40.0%) and 27 females with a mean age of 47.4 years (range 21-73); 40 (88.9%) self-identified as being from a ‘white’ background and 35 (77.9%) were in employment/ education/ training. There were no significant differences between those that provided belief flexibility data compared to those that did not on any key demographic or JTC reasoning variable.

**Additional Exploratory analysis:**

*Table 1:* Associations between use of rational reasoning (RR), experiential reasoning (ER), and both styles of reasoning and JTC and (Main) belief flexibility items

|  | RR max | RR not max | Significance tests | Odds Ratios (95% CI) |
| --- | --- | --- | --- | --- |
| JTC (≤2) (%) | 11 (50) | 11 (50) | χ^2^ (1)=3.339 , p=.068 | 2.417 (0.924, 6.322) |
| No JTC (%) | 58 (70.7) | 24 (29.3) |
|  | ER max | ER not max |  |  |
| JTC (≤2) (%) | 12 (54.5) | 10 (45.5) | χ^2^ (1)=0.031 , p=.860 | 0.919 (0.357, 2.363) |
| No JTC (%) | 43 (52.4) | 39 (47.6) |
|  | RR & ER max | Not max |  |  |
| JTC (≤2) n (%) | 5 (22.7) | 17 (77.3) | χ^2^ (1)=0.669 , p=.414 | 1.579 (0.525, 4.744) |
| No JTC (%) | 26 (31.7) | 56 (68.3) |
|  | RR max | RR not max |  |  |
| PM (Main)-YES (%) | 46 (74.2) | 16 (25.8) | **χ^2^ (1)=4.234, p=.040** | **2.375 (1.033, 5.459)** |
| PM NO (%) | 23 (54.8) | 19 (45.2) |
|  | ER max | ER not max |  |  |
| PM (Main)-YES (%) | 29 (46.8) | 33 (53.2) | χ^2^ (1)=2.301, p=.129 | 0.541 (0.243, 1.201) |
| PM NO (%) | 26 (61.9) | 16 (38.1) |
|  | RR & ER max | Not max |  |  |
| PM (Main)-YES (%) | 19 (30.6) | 43 (69.4) | χ^2^ (1)=0.051 , p=.821 | 1.105 (0.467, 2.611) |
| PM NO (%) | 12 (28.6) | 30 (71.4) |
|  | RR max | RR not max |  |  |
| AE (Main)-YES (%) | 48 (76.2) | 15 (23.8) | **χ^2^ (1)=6.936, p=.008** | **3.048 (1.311, 7.083)** |
| AE NO (%) | 21 (51.2) | 20 (48.8) |
|  | ER max | ER not max |  |  |
| AE (Main)-YES (%) | 30 (47.6) | 33 (52.4) | χ^2^ (1)=1.778 , p=.182 | 0.582 (0.262, 1.293) |
| AE NO (%) | 25 (61) | 16 (39) |
|  | RR & ER max | Not max |  |  |
| AE (Main)-YES (%) | 20 (31.7) | 43 (68.3) | χ^2^ (1)=0.287 , p=.592 | 1.268 (0.531, 3.031) |
| AE NO (%) | 11 (26.8) | 30 (73.2) |

**Additional analysis of IQ and key reasoning variables.**

Clinical and non-clinical individuals show differences in a range of demographic and clinical variables, which are inherent to their group status. For example Ward and colleagues^24^ reported differences in onset age of experiences, marital status, employment, IQ, ethnicity, current anxiety, and depression, all favouring the non-clinical group. These naturally occurring group differences include variables that reflect established risk factors for need-for-care, such as low IQ/poorer pre-morbid functioning and ethnicity^33-35^, and/or are sequelae of group status (e.g. impaired functioning, social isolation and anxiety). When comparing groups to examine differences in hypothesised variables, it is not always desirable to ‘control for’ other group differences, which may be related to the variables of interest (e.g. IQ, which is likely to be related to specific reasoning biases). As such the main paper reports the group differences on our hypothesised variables without including as covariates in the analysis established risk factors for psychosis, on which the groups differ (such as IQ, ethnicity and gender) or those inherent to need-for-care status (e.g. impaired functioning, anxiety, depression etc). The following are therefore presented for the reader’s reference with this important caveat.

*Table 2. Linear regression for ‘Beads Drawn’ (Dependent Variable) with Group (dummy coded) and IQ entered as predictor variables*

|  | B | SE (B) | β | p-values |
| --- | --- | --- | --- | --- |
| Constant | 1.52 | 2.60 |  |  |
| Control vs. Clinical | 2.54 | 1.22 | .187 | P=.039 |
| Non-clinical vs. Clinical | 1.94 | 1.11 | .145 | p=.082 |
| IQ | 0.064 | 0.03 | .171 | p=.023 |

R^2^=.085 (p<.001)

*Table 3: Binary logistic regression for JTC Extreme responding’ (Dependent Variable) with Group and IQ entered as predictor variables*

| **Variables in the Equation** | | | | | | | | | |
| --- | --- | --- | --- | --- | --- | --- | --- | --- | --- |
|  | | B | S.E. | Wald | df | Sig. | Exp(B) | 95% C.I.for EXP(B) | |
|  |  |  |  |  |  |  |  | Lower | Upper |
| Step 1^a^ | Overall_group |  |  | 1.231 | 2 | .540 |  |  |  |
|  | Control versus Clinical | .537 | .506 | 1.130 | 1 | .288 | 1.712 | .635 | 4.611 |
|  | Non-Clinical vs. Clinical | .124 | .428 | .084 | 1 | .772 | 1.132 | .489 | 2.620 |
|  | WAIS_estimated_IQ | .047 | .014 | 11.634 | 1 | .001 | 1.049 | 1.020 | 1.078 |
|  | Constant | -3.549 | 1.232 | 8.300 | 1 | .004 | .029 |  |  |
| Model χ^2^(3)=26.523, p<.001, R^2^ (Cox and Snell= .105, Nagelkerke = .163). | | | | | | | | | |

*Table 4: Binary logistic regression for Possibility of being mistake (Dependent Variable) with Group and IQ entered as predictor variables*

| **Variables in the Equation** | | | | | | | | | |
| --- | --- | --- | --- | --- | --- | --- | --- | --- | --- |
|  | | B | S.E. | Wald | df | Sig. | Exp(B) | 95% C.I.for EXP(B) | |
|  |  |  |  |  |  |  |  | Lower | Upper |
| Step 1^a^ | Overall_group |  |  | 4.210 | 2 | .122 |  |  |  |
|  | Control versus Clinical | .527 | .512 | 1.058 | 1 | .304 | 1.694 | .621 | 4.623 |
|  | Non-Clinical vs. Clinical | -.316 | .372 | .721 | 1 | .396 | .729 | .351 | 1.512 |
|  | WAIS_estimated_IQ | .005 | .011 | .246 | 1 | .620 | 1.005 | .984 | 1.027 |
|  | Constant | -.200 | .979 | .042 | 1 | .838 | .819 |  |  |
| Model χ^2^(3)=5.155, p=.161, R^2^ (Cox and Snell= .026, Nagelkerke = .035). | | | | | | | | | |

*Table 5: Binary logistic regression for Alternative Explanations (Dependent Variable) with Group and IQ entered as predictor variables*

| **Variables in the Equation** | | | | | | | | | |
| --- | --- | --- | --- | --- | --- | --- | --- | --- | --- |
|  | | B | S.E. | Wald | df | Sig. | Exp(B) | 95% C.I.for EXP(B) | |
|  |  |  |  |  |  |  |  | Lower | Upper |
| Step 1^a^ | Overall_group |  |  | .495 | 2 | .781 |  |  |  |
|  | Control versus Clinical | .072 | .512 | .020 | 1 | .889 | 1.074 | .394 | 2.932 |
|  | Non-Clinical vs. Clinical | -.183 | .380 | .231 | 1 | .631 | .833 | .395 | 1.755 |
|  | WAIS_estimated_IQ | .030 | .011 | 6.762 | 1 | .009 | 1.030 | 1.007 | 1.054 |
|  | Constant | -2.632 | 1.041 | 6.397 | 1 | .011 | .072 |  |  |

Model χ^2^(3)=11.025, p=.012, R^2^ (Cox and Snell= .055, Nagelkerke = .074).

*Table 6: Binary logistic regression for Possibility of being Mistaken (Control Belief) (Dependent Variable) with Group and IQ entered as predictor variables*

| **Variables in the Equation** | | | | | | | | | |
| --- | --- | --- | --- | --- | --- | --- | --- | --- | --- |
|  | | B | S.E. | Wald | df | Sig. | Exp(B) | 95% C.I.for EXP(B) | |
|  |  |  |  |  |  |  |  | Lower | Upper |
| Step 1^a^ | Overall_group |  |  | 2.143 | 2 | .343 |  |  |  |
|  | Control versus Clinical | -.651 | .496 | 1.721 | 1 | .190 | .522 | .197 | 1.379 |
|  | Non-Clinical vs. Clinical | -.505 | .381 | 1.755 | 1 | .185 | .604 | .286 | 1.274 |
|  | WAIS_estimated_IQ | .034 | .011 | 9.161 | 1 | .002 | 1.035 | 1.012 | 1.058 |
|  | Constant | -3.085 | 1.028 | 8.999 | 1 | .003 | .046 |  |  |
|  | | | | | | | | | |
| Model χ^2^(3)=10.385, p=.016; R^2^ (Cox and Snell= .050, Nagelkerke = .067). | | | | | | | | | |

*Table 7: Binary logistic regression for Rational reasoning (Dependent Variable) with Group and IQ entered as predictor variables*

| **Variables in the Equation** | | | | | | | | | |
| --- | --- | --- | --- | --- | --- | --- | --- | --- | --- |
|  | | B | S.E. | Wald | df | Sig. | Exp(B) | 95% C.I.for EXP(B) | |
|  |  |  |  |  |  |  |  | Lower | Upper |
| Step 1^a^ | Overall_group |  |  | 7.345 | 2 | .025 |  |  |  |
|  | Control versus Clinical | 1.609 | .698 | 5.320 | 1 | .021 | 4.999 | 1.274 | 19.620 |
|  | Non-Clinical vs. Clinical | 1.581 | .638 | 6.134 | 1 | .013 | 4.860 | 1.391 | 16.983 |
|  | WAIS_estimated_IQ | .018 | .017 | 1.155 | 1 | .282 | 1.018 | .985 | 1.053 |
|  | Constant | -2.099 | 1.546 | 1.842 | 1 | .175 | .123 |  |  |
| Model χ^2^(3)=18.611, p<.001, R^2^ (Cox and Snell= .171, Nagelkerke = .241). | | | | | | | | | |

*Table 8: Binary logistic regression for Experiential reasoning (Dependent Variable) with Group and IQ entered as predictor variables*

| **Variables in the Equation** | | | | | | | | | |
| --- | --- | --- | --- | --- | --- | --- | --- | --- | --- |
|  | | B | S.E. | Wald | df | Sig. | Exp(B) | 95% C.I.for EXP(B) | |
|  |  |  |  |  |  |  |  | Lower | Upper |
| Step 1^a^ | Overall_group |  |  | 25.784 | 2 | .000 |  |  |  |
|  | Control versus Clinical | -1.179 | .704 | 2.804 | 1 | .094 | .308 | .077 | 1.223 |
|  | Non-Clinical vs. Clinical | 2.151 | .719 | 8.957 | 1 | .003 | 8.592 | 2.101 | 35.144 |
|  | WAIS_estimated_IQ | -.012 | .017 | .515 | 1 | .473 | .988 | .955 | 1.021 |
|  | Constant | .998 | 1.532 | .424 | 1 | .515 | 2.714 |  |  |
| Model χ^2^(3)=36.798, p<.001, R^2^ (Cox and Snell= .310, Nagelkerke = .414). | | | | | | | | | |

*Table 9: Binary logistic regression for ‘Evidence of Experiential and Rational Reasoning’ (Dependent Variable) with Group and IQ entered as predictor variables*

| **Variables in the Equation** | | | | | | | | | |
| --- | --- | --- | --- | --- | --- | --- | --- | --- | --- |
|  | | B | S.E. | Wald | df | Sig. | Exp(B) | 95% C.I.for EXP(B) | |
|  |  |  |  |  |  |  |  | Lower | Upper |
| Step 1^a^ | Overall_group |  |  | 26.905 | 2 | .000 |  |  |  |
|  | Control versus Clinical | -.906 | 1.071 | .716 | 1 | .397 | .404 | .050 | 3.295 |
|  | Non-Clinical vs. Clinical | 2.638 | .779 | 11.477 | 1 | .001 | 13.988 | 3.040 | 64.365 |
|  | WAIS_estimated_IQ | .011 | .019 | .373 | 1 | .541 | 1.012 | .975 | 1.049 |
|  | Constant | -3.170 | 1.774 | 3.192 | 1 | .074 | .042 |  |  |
| Model χ^2^(3)=39.787, p<.001, R^2^ (Cox and Snell= .331, Nagelkerke = .472). | | | | | | | | | |

**References**

Bebbington, P., & Nayani, T. (1995). The Psychosis Screening Questionnaire. *International Journal of Methods in Psychiatric Research, 5*(1), 11-19.

Brett, C. M., Peters, E. P., Johns, L. C., Tabraham, P., Valmaggia, L. R., & McGuire, P. (2007). Appraisals of Anomalous Experiences Interview (AANEX): a multidimensional measure of psychological responses to anomalies associated with psychosis. *Br J Psychiatry Suppl, 51*, s23-30.

Hatch, S. L., Frissa, S., Verdecchia, M., Stewart, R., Fear, N. T., Reichenberg, A., . . . Team, S. S. (2011). Identifying socio-demographic and socioeconomic determinants of health inequalities in a diverse London community: the South East London Community Health (SELCoH) study. *Bmc Public Health,* 11.

Heriot-Maitland, C., Knight, M., & Peters, E. (2012). A qualitative comparison of psychotic-like phenomena in clinical and non-clinical populations. *Br J Clin Psychol, 51*(1), 37-53.

Mason, O., & Claridge, G. (2006). The Oxford-Liverpool Inventory of Feelings and Experiences (O-LIFE): Further description and extended norms. *Schizophrenia Research, 82*(2-3), 203-211.

Peters, E., Ward, T., Jackson, M., Morgan, C., Charalambides, M., McGuire, P., Woodruff, P.,

Jacobsen, P., Chadwick, P., & Garety, P. (2016). Clinical, socio-demographic and psychological characteristics in individuals with persistent psychotic experiences with and without a “need for care”. *World Psychiatry, 15(1),* 41-52.

Ward, T. A., Gaynor, K. J., Hunter, M. D., Woodruff, P. W., Garety, P. A., & Peters, E. R. (2014). Appraisals and responses to experimental symptom analogues in clinical and nonclinical individuals with psychotic experiences. *Schizophr Bull, 40*(4), 845-855.
